# EHMTI-0126. Superiority of algopirin® versus excedrin® in treating migraine. Individual pain values and pain curves comparisons

**DOI:** 10.1186/1129-2377-15-S1-G25

**Published:** 2014-09-18

**Authors:** C Mircioiu, V Voicu, I Mircioiu

**Affiliations:** 1 Biostatistics, UMF Carol Davila, Bucharest, Romania; 2 Clinical Pharmmacology and Toxicology, UMF Carol Davila, Bucharest, Romania; 3 Biopharmacy, Ovidius University, Constanta, Romania

## Background

A synergic association between acetylsalycilic acid, acetaminophen, caffeine in doses 3-4 times lower than similar combinations and chlorpheniramine was patented and marketed as analgesic drug. A previous study proved the non-inferiority of a unique dose of treatment using Algopirin®, versus Excedrin® similar association.

Aim of the research was to prove superiority of two tablets Algopirin® versus one tablet Excedrin® in treating migraine.

## Method

Patients treated two independent migraine episodes of at least moderate intensity with Algopirin® in first period and with Excedrin® (Novartis) in second period. All patients recorded the pain severity on a Visual Analog Scale before and 30, 60, 120, 180 and 240 min after drug intake. Comparison of the mean pain intensities was performed using paired t test and Wilcoxon pair signed rank test. Interpretation of pain curves as “survival pain curves” allowed application of statistical methods usual in cancer research. Comparison of areas under curves was performed similar with comparison of plasma levels of drugs in pharmacokinetics.

## Results

Effect of Algopirin® was greater than that of Excedrin®. Mean time to 50 % (T50) pain relief was 30 min for Algopirin® and 45 min for Excedrin. Time to pain relief to 20 % (T20) was 85 min for Algopirin® and 180 min for Excedrin®. Difference between effects measured by areas under curves was 11%. All differences were statistically significant (p<0.01).

## Conclusions

In spite of the fact that Algopirin® contains lower doses of active components, its effect on pain curves in migraine is superior to Excedrin®.

No conflict of interest.

